# A Novel Model Based on Spatial and Morphological Domains to Predict the Origin of Premature Ventricular Contraction

**DOI:** 10.3389/fphys.2021.641358

**Published:** 2021-02-24

**Authors:** Kaiyue He, Jian Sun, Yiwen Wang, Gaoyan Zhong, Cuiwei Yang

**Affiliations:** ^1^Department of Electronic Engineering, Fudan University, Shanghai, China; ^2^Department of Cardiology, School of Medicine, Xinhua Hospital, Shanghai Jiao Tong University, Shanghai, China; ^3^Key Laboratory of Medical Imaging Computing and Computer Assisted Intervention of Shanghai, Shanghai Medical College of Fudan University, Shanghai, China; ^4^Shanghai Engineering Research Center of Cardiac Electrophysiology, Shanghai, China

**Keywords:** pace mapping, ventricular arrhythmias, ablation, automated algorithm, origin of PVC

## Abstract

Pace mapping is commonly used to locate the origin of ventricular arrhythmias, especially premature ventricular contraction (PVC). However, this technique relies on clinicians’ ability to rapidly interpret ECG data. To avoid time-consuming interpretation of ECG morphology, some automated algorithms or computational models have been explored to guide the ablation. Inspired by these studies, we propose a novel model based on spatial and morphological domains. The purpose of this study is to assess this model and compare it with three existing models. The data are available from the Experimental Data and Geometric Analysis Repository database in which three *in vivo* PVC patients are included. To measure the hit rate (A hit occurs when the predicted site is within 15 mm of the target) of different algorithms, 47 target sites are tested. Moreover, to evaluate the efficiency of different models in narrowing down the target range, 54 targets are verified. As a result, the proposed algorithm achieves the most hits (37/47) and fewest misses (9/47), and it narrows down the target range most, from 27.62 ± 3.47 mm to 10.72 ± 9.58 mm among 54 target sites. It is expected to be applied in the real-time prediction of the origin of ventricular activation to guide the clinician toward the target site.

## Introduction

Premature ventricular contraction (PVC) is one of the most common ventricular arrhythmias encountered in clinical practice, occurring in 1–4% of the general population (Kostis et al., 1981). Frequent and repetitive PVCs can increase the risk of arrhythmia-induced syncope, ventricular dysfunction, and sudden death (Ahn, 2013). Hitherto, catheter ablation has become an important therapy in the management of ventricular arrhythmias (Al-Khatib et al., 2018). In the last decade (from 2000 to 2012), the annual ventricular tachycardia (VT) ablation volumes have quadrupled (Hsia and Xiong, 2019). Also, multiple studies have shown that catheter ablation can be more effective in reducing arrhythmia recurrence than anti-arrhythmic drugs (Sapp et al., 2016).

It is of therapeutical importance to localize the origin of abnormal ventricular activation before catheter ablation. The localization can be done by several approaches. Activation mapping is the most direct technique which can be applied in patients with frequent PVCs (Adams et al., 2012). Yet, it requires time-consuming intracardiac mapping by moving the catheter to different sites of the ventricles, and it can only be performed in a small number of patients who can endure a sustained VT during the whole mapping operation. Since the origin of PVC largely determines the QRS morphology of 12-lead ECG (Josephson et al., 1982), an alternative technique, known as pace mapping, can be applied in more patients by physically stimulating multiple ventricle sites until finding the site where pacing reproduces the morphology of spontaneous PVC (Kobayashi, 2018). However, this practice relies heavily on rapid and accurate manual interpretation of ECG.

In order to automatically analyze the information of pacing sites and progressively guide the clinician to the origin of PVC, several methods have been developed. One method is to train a universal model from a cohort of patients based on machine learning methods (Sapp et al., 2017; Zhou et al., 2019). However, due to anatomical and physiological variations in patients, there is a limited accuracy when a universal model is applied to a new patient. An alternative strategy is to build patient-specific prediction models. To our knowledge, some studies have used the image-based simulated ECG data to train a customized prediction model for each patient (Potse et al., 2000; Yang et al., 2018) and the domain adaptation method has newly been applied to modify the prediction model with clinical data to account for the potential errors in the simulation data (Alawad and Wang, 2019).

In addition to the image-based simulation method, some simpler but less computational models based on information of multiple pacing sites have also been investigated. Lately, the QRS integrals (QRS-Ints) of 12-lead ECG have been used to predict the 3D coordinate of the PVC origin directly (Sapp et al., 2017; Zhou et al., 2018). Besides, the relationship between distance and change in 12-lead ECG morphology has also been inspected to assist in the localization of PVC origin (Li et al., 2017, 2018; Odille et al., 2019; Dharmaprani et al., 2020).

Inspired by previous studies, in this paper, a novel model only based on the information of pacing sites is proposed and compared with three existing models [QRS-Int Model (Sapp et al., 2017), dis-E12 Model (Li et al., 2017), and dis-corr Model (Dharmaprani et al., 2020)]. We evaluated these models in three patients with PVC and found that the proposed model was slightly superior to the other three models. This method is very suitable for the location of PVC origins in non-organic heart disease.

## Materials and Methods

### Data

The data used throughout this study is obtained from the Experimental Data and Geometric Analysis Repository (EDGAR) database (Aras et al., 2015). The data were collected during endocardial pacing from three PVC patients. The patients were consented for an add-on experimental procedure involving ventricular pacing, performed according to a protocol approved by the ethical committee of Charles University Hospital, Prague, Czechia (Erem et al., 2014). For each patient, there is a mean of 25 ± 6 distinct sites of endocardial pacing with known coordinates. For each pacing site, a mean of 28 ± 8 ECG beats are available and a representative beat is calculated by averaging these beats. The equation is as follows:

(1) $$V_{i}=\frac{1}{N}\,\sum_{n=1}^{N}V_{i}^{\left(n\right)}$$

where *V_i* and $V_{i}^{\left(n\right)}$ are the *i*th-lead ECG signals of representative beat and beat n, respectively.

### Models

#### QRS-Int Model

The QRS-Int values were proposed by Sapp et al. (2017) as predictor variables to fit the geometric coordinate system of the heart. A statistical estimate of the coordinates $\hat{x}$, $\hat{y}$, and $\hat{z}$ for any pacing site can be obtained by fitting the multiple linear regression equation with intercept. The equation is as follows:

(2) $$\left[\begin{array}{c} \hat{\text{x}}\\ \hat{\text{y}}\\ \hat{\text{z}} \end{array}\right]=\left[\begin{array}{cc} \begin{array}{ccc} \hat{\alpha_{0}} & \hat{\alpha_{1}} & \cdots \end{array} & \hat{\alpha_{k}}\\ \begin{array}{ccc} \hat{\beta_{0}} & \hat{\beta_{1}} & \cdots \end{array} & \hat{\beta_{k}}\\ \begin{array}{ccc} \hat{\gamma_{0}} & \hat{\gamma_{1}} & \cdots \end{array} & \hat{\gamma_{k}} \end{array}\right]\,\left[\begin{array}{c} 1\\ {\text{I}}_{1}\\ \begin{array}{c} \cdots \\ {\text{I}}_{k} \end{array} \end{array}\right]$$

where $\hat{\alpha_{i}}$, $\hat{\beta_{i}}$, and $\hat{\gamma_{i}}$ are estimated regression coefficients, and *I_i* represents the QRS-Int. To minimize the training set of required pacing sites, three optimal predictors (the initial 120-ms QRS-Int of leads III, V2, and V6) were found by exhaustive search (Sapp et al., 2017). Then, the least-square method was used to solve 4 equations (*k* = 3) to obtain the patient-specific QRS integral model (QIM). We used at least 5 pacing points to avoid matrix singularity. The best regression coefficients can be calculated by least-square regression (Sapp et al., 2017). Once the regression coefficients best fitted for the training-set data are found, they can be used for prediction of the unknown site. Here, the initial 120-ms QRS-Int values are extracted manually from the representative beat of each pacing site.

#### dis-E12 Model

The E12 value proposed by Anthony et al. (Li et al., 2017) can be used to quantify the difference of 12-lead ECG between 2 pacing sites.

$$E_{12}=\sum_{i=1}^{12}\sqrt{\frac{1}{N}\,\sum_{j=1}^{N}{\left(V_{i,j}-V_{i,j}^{r}\right)}^{2}}$$

(3) $$d\,i\,s=\sqrt{{\left(x-x^{r}\right)}^{2}+{\left(y-y^{r}\right)}^{2}+{\left(z-z^{r}\right)}^{2}}$$

$$d\,i\,s=k_{1}\cdot E_{12}$$

$$\hat{d\,i\,s^{i}}=k_{1}\cdot E_{12}\,\left(V^{i\,t\,h-k\,n\,o\,w\,n},V^{u\,n\,k\,n\,o\,w\,n}\right)$$

where V and *V*^*r*^ represent 2 pacing beats being compared, which are 150-ms waveforms centered on the maximum of the 12-lead composite signal (Li et al., 2017), and **V**_**i**,**j**_ and ${\text{V}}_{i,j}^{r}$ represent the voltages of one moment of the ECG. *N* is the length of the ECG signal. *i* ranges from 1 to 12, representing the index of 12 leads. *dis* represents the Euclidean distance between pacing sites. Similarly, the patient-specific dis-E12 model (DEM) can be solved by origin-constrained least-square linear regression. After that, the E12 value between the unknown site and each known site is calculated and then used to estimate the corresponding distance $\hat{d\,i\,s^{i}}$ for i1,2,⋯,m. Finally, by minimizing the following cost function *J*, a statistical estimate of coordinates $\hat{x}$, $\hat{y}$, and $\hat{z}$ for the unknown site can be found.

(4) $$J=\sum_{i=1}^{m}(\sqrt{{\left(\hat{x}-x_{i}\right)}^{2}+{\left(\hat{y}-y_{i}\right)}^{2}+{\left(\hat{z}-z_{i}\right)}^{2}-\hat{d\,i\,s^{i}}){}^{2}}$$

#### dis-Corr Model

The correlation coefficient (Corr) proposed by Dharmaprani et al. (2020) can be used to quantify the similarity of ECG morphology between 2 pacing sites.

$$r\,\left(X,Y\right)=\frac{\sum_{i=1}^{n}\left(X_{i}-\bar{X}\right)\,\left(Y_{i}-\bar{Y}\right)}{\sqrt{\sum_{i=1}^{n}{\left(X_{i}-\bar{X}\right)}^{2}}\,\sqrt{\sum_{i=1}^{n}{\left(Y_{i}-\bar{Y}\right)}^{2}}}$$

$$C\,o\,r\,r=\frac{1}{12}\,\sum_{i=1}^{12}r\,\left(V_{i},V_{i}^{r}\right)$$

(5) $$d\,i\,s=\sqrt{{\left(x-x^{r}\right)}^{2}+{\left(y-y^{r}\right)}^{2}+{\left(z-z^{r}\right)}^{2}}$$

$$d\,i\,s=k_{2}\cdot \left(C\,o\,r\,r-1\right)$$

$$\hat{d\,i\,s^{i}}=k_{2}\cdot \left(1-C\,o\,r\,r\,\left(V^{i\,t\,h-k\,n\,o\,w\,n},V^{u\,n\,k\,n\,o\,w\,n}\right)\right)$$

where *r*(*X*,*Y*) represents the Pearson correlation coefficient between time series X and Y. Moreover, the Corr value is the average result of 12 leads. Similarly, the patient specific dis-Corr model (DCM) can be solved by constrained least-square linear regression, and the Corr value between the unknown site and each known site can also be calculated and transformed into the estimated distance $\hat{d\,i\,s^{i}}$ for i1,2,⋯,m. Finally, by minimizing the cost function *J* presented in Eq. (4), a statistical estimate of coordinates $\hat{x}$, $\hat{y}$, and $\hat{z}$ for the unknown site can be found.

#### dp-dw Model

**FIGURE 1:**
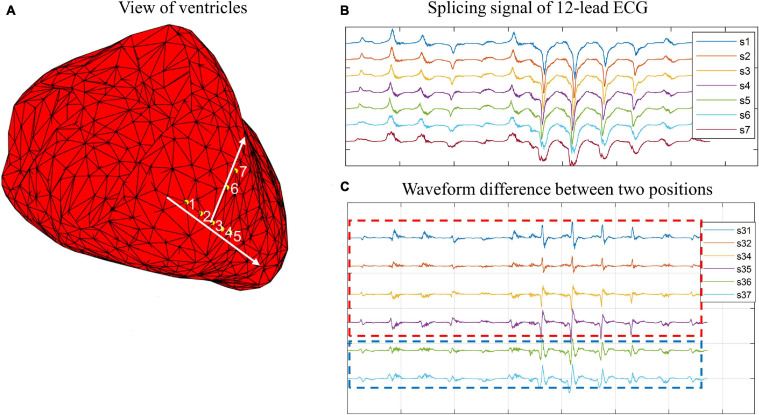
Example of connections between wave morphology and physical positions on simulation data. **(A)** View of ventricles. Yellow points 1, 2, 3, 4, 5, 6, and 7 are simulated paced points. Points 1, 2, 3, 4, and 5 are almost on the same line, while point 5, 6, and 7 are almost on another line. **(B)** Splicing signal of 12-lead ECG. The splicing signals are one-dimensional vectors formed by stitching 12 time-series (each of 150-ms) of corresponding points. **(C)** Waveform difference between two points. The waveform differences are extracted by subtracting one waveform vector from another waveform vector.

**FIGURE 2:**
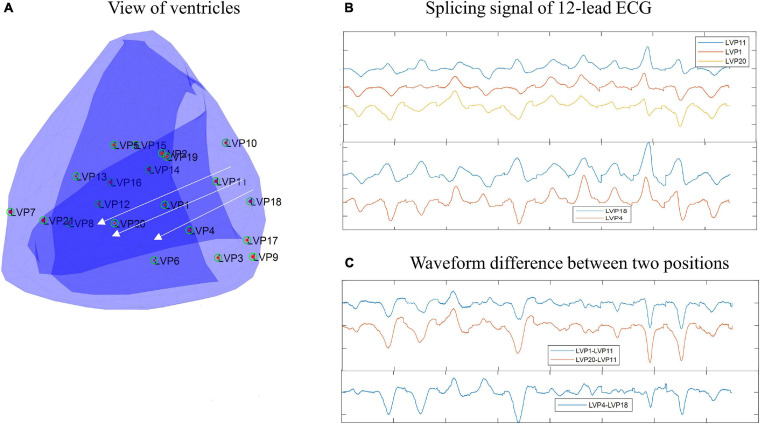
Example of connections between wave morphology and physical positions on real data. **(A)** View of ventricles. The points are paced points. **(B)** Splicing signal of 12-lead ECG. The splicing signals are one-dimensional vectors formed by stitching 12 time-series (each of 150-ms) of corresponding points. **(C)** Waveform difference between two points. The waveform differences are extracted by subtracting one waveform vector from another waveform vector.

In this study, we observed a phenomenon that there are some connections between waveform morphology and physical position. Figures 1, 2 show two examples based on simulation data and real data, respectively. The simulation data were generated by an isotropic ventricular simulation model with electric conduction rate of 0.7 m/s (Schulze et al., 2015). As can be observed from Figure 1, points 1, 2, 3, 4, and 5 are almost on the same line, while points 3, 6, and 7 are almost on another line. Figure 1B shows the splicing signal of 12-lead ECG in accordance with positions, and Figure 1C shows the waveform difference between each pair of positions. It seems that the waveform differences on the same line are more similar, while those on different lines are less similar. For example, s31, s32, s34, and s35 are similar with each other and so are s36 and s37, but s31 and s37 are less similar. Then the real data are extracted from the first patient. As can be seen from Figure 2, points LVP11, LVP1, and LVP20 are almost on the same line, while LVP18 and LVP4 are almost on another line. Besides, the two lines are nearly parallel. Similarly, we observed the similarity of waveform differences on the same line. We also observed the similarity of waveform differences between parallel lines.

The above phenomenon may be explained by the theory of electrocardiographic dipoles. During ventricular depolarization, electric dipoles can be formed between depolarized and non-depolarized regions, and the integrated vector of all dipoles can be recorded by 12 leads from different positions and directions. The recorded voltage on each lead at one moment is related to the distances between the recording electrode and the electric dipoles, and it is also related to the cosine angles formed by the orientation of the lead axis and the directions of myocardial depolarization. When a ventricular premature occurs, the depolarization wave spreads from the earliest excitation point to all sides, and the directions of electric dipoles are the same as the directions of myocardial depolarization. When the earliest excitation point moves along a certain direction, the electric dipoles will change most in the same direction, which may lead to more obvious waveform changes in leads parallel to the direction and less obvious waveform changes in leads perpendicular to the direction. Therefore, there might be a certain relationship between the waveform changes of 12-lead ECG and the position changes of the earliest excitation point.

**FIGURE 3:**
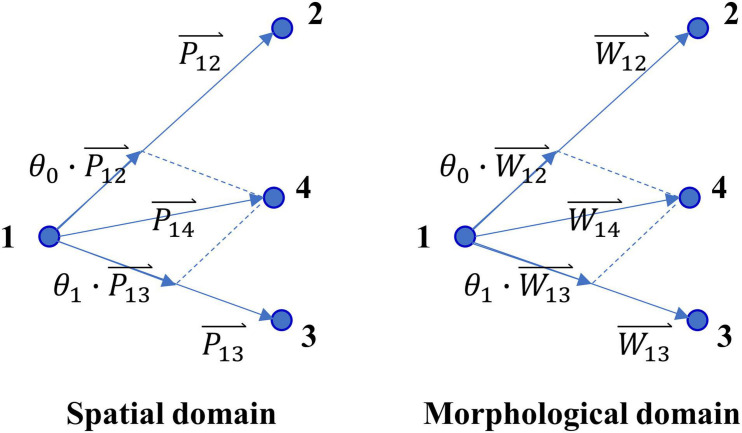
Diagram of the counterpart connections between spatial domain and morphological domain.

Based on these observations, we proposed a novel prediction model based on the assumption that there are some counterpart connections between the spatial domain and the morphological domain. As Figure 3 shows, Pi⁢j⇀i⁢j and Wi⁢j⇀i⁢j represent the vector of position difference (dp) and the vector of waveform difference (dw) between point i and point j, respectively. Here, ${\vec{W}}_{i}$ represents a one-dimensional vector formed by stitching 12 time-series (each of 150-ms) of pacing site i together, and Wi⁢j⇀i⁢j is obtained by subtracting ${\vec{W}}_{i}$ from ${\vec{W}}_{j}$. Supposing Eq. (6) holds in the spatial domain, and then Eq. (7) holds in the morphological domain, and vice versa.

(6) P14⇀14=θ0⋅P12⇀12+θ1⋅P13⇀13

(7) W14⇀14=θ0⋅W12⇀12+θ1⋅W13⇀13

**FIGURE 4:**
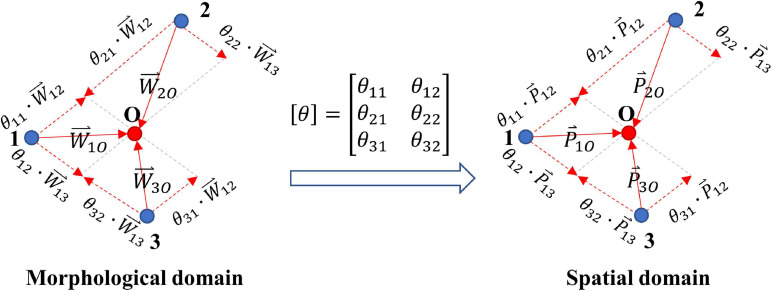
Establishment and prediction process of DDM.

Figure 4 illustrates the establishment and prediction process of the dp-dw model (DDM). As shown in the figure, points 1, 2, and 3 are the known sites whose 12-lead ECG signals and physical locations are known by us, while point O is the unknown site whose 12-lead ECG information is known by us, but its physical location needs to be estimated by algorithm. Of course, the actual physical location of point O is known, but we pretend not to know that. Moreover, we use the waveform difference between point O and its adjacent points to estimate its location. The vectors of dp and dw between each pair of known sites are calculated as $\left[\Delta P\right]\;\;\left[\vec{P_{12}},\vec{P_{13}}\right]$ and $\left[\Delta W\right]\;\;\left[\vec{W_{12}},\vec{W_{13}}\right]$, and the vector of dw between the unknown site and each known site is calculated as [△⁢W*]⁢[W1⁢O⇀1⁢O,W2⁢O⇀2⁢O,W3⁢O⇀3⁢O]. Analogously, the transfer matrix [θ] mapping [△*W*] to $\left[\hat{△\,W^{*}}\right]$ can be found by least-square regression [Eq. (8)]. Then, by applying the same transfer matrix [θ] to the spatial domain, the estimated vector of dp between the unknown site and each known site can be calculated as shown in Eq. (9). Finally, a statistical estimate of coordinates $\hat{x}$, $\hat{y}$, and $\hat{z}$ for the unknown site can be calculated as Eq. (10).

$$\left[\hat{△\,W^{*}}\right]=\left[△\,W\right]\times \left[\theta \right]$$

(8) $$={\left({\left[△\,W\right]}^{T}\times \left[△\,W\right]\right)}^{-1}\times {\left[△\,W\right]}^{T}\times \left[△\,W^{*}\right]$$

=[△⁢P]×[θ]

(9) =[P1⁢O⇀1⁢O^,P2⁢O⇀^,P3⁢O⇀^]

(10) $$\left[\begin{array}{c} \hat{x}\\ \hat{y}\\ \hat{z} \end{array}\right]=\frac{1}{3}\,\sum_{i=1}^{3}\left(P_{i}+\hat{\overset{⇀}{\underset{i\,O}{P}}}\right)$$

**FIGURE 5:**
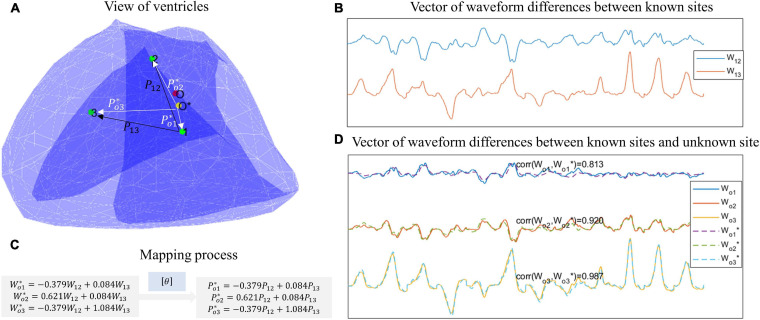
An example of using DDM to predict the PVC origin. **(A)** View of ventricles. The green points represent known sites, the red point represents the unknown site, and the yellow site represents the predicted site. **(B)** Vector of waveform differences between known sites. For example, W12 is extracted by subtracting the waveform of point 1 from the waveform of point 2. **(C)** Mapping process. By least-square regression, the estimated waveform differences between the known sites and the unknown sites can be transformed from the waveform differences between the known sites. Then by applying the same transfer matrix in the spatial domain, the position differences between the known sites and the unknown site can be calculated. **(D)** Vector of waveform difference between known sites and unknown site. The ground-truth waveform differences are shown with solid lines, while the estimated waveform differences are shown with dotted lines.

Figure 5 shows an example of using DDM to predict the PVC origin. In Figure 5A, the red point represents the unknown site, the green points represent the known sites, and the yellow point represents the predicted position. Figure 5B shows the waveform differences (dws) between the known sites, and Figure 5D shows the dws between the known sites and the unknown site. By using least-square regression, the estimated dws between the known sites and the unknown site can be transformed from the dws between the known sites, as Figure 5C shows. Finally, by applying the same transfer matrix [θ]in the spatial domain, the position differences (dps) between the known sites and the unknown site could be calculated. And by executing Eq.(10), the predicted position was obtained.

### Emulation of Clinical Protocols

#### Target Site Selection

Two target site selection schemes were adopted for different purposes. First, in order to evaluate the hit rate (a hit occurs when the predicted site is within 15 mm of the target), the target site is defined as the site with at least 5 adjacent sites which are greater than 15 mm and less than 35 mm away from it, and a total of 47 pacing sites meet the conditions. Secondly, in order to evaluate the efficiency of different models in narrowing down the target range, the target site is defined having at least 5 adjacent sites within the range of 35 mm of it, and a total of 54 pacing sites meet the requirement. Once a target site is selected, its adjacent sites that meet the corresponding definition serve as potential modeling sites. In this study, the modeling sites are those whose physical locations and corresponding 12-lead ECG signals are known, while the target sites are those whose 12-lead ECG signals are known, but their physical locations need to be estimated by the algorithm.

### Modeling and Prediction

**FIGURE 6:**
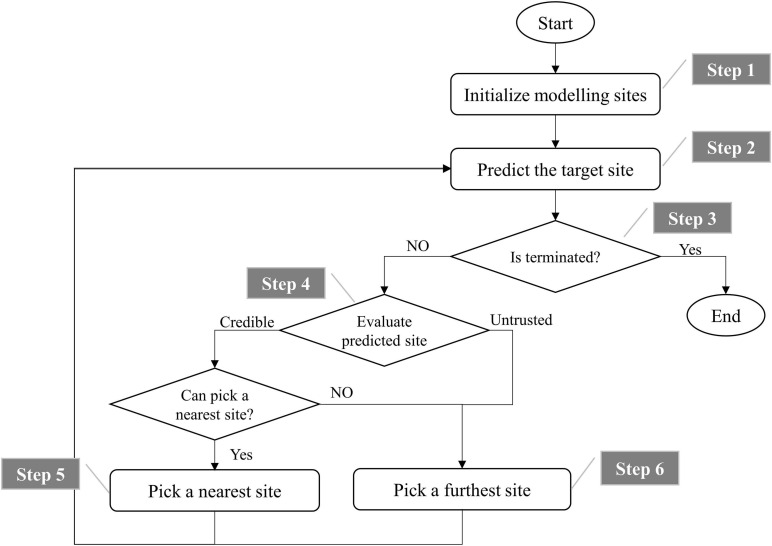
Flowchart of modeling and prediction.

Figure 6 shows the flowchart of the process of modeling and prediction. It can be divided into the following six steps.

Step 1: Initialize modeling sites. The 3 or 5 farthest unused potential modeling sites (3 for DEM, DCM, and DDM, and 5 for QIM) from the target site are selected as initial modeling sites, and they will be removed from the list of unused potential modeling sites. For example, supposing point 0 is selected as the target site, and its adjacent points 1, 2, 3, 4, 5, and 6 match the definition of potential modeling sites. Therefore, the initial list of unused potential modeling sites is (Kostis et al., 1981; Adams et al., 2012; Ahn, 2013; Sapp et al., 2016; Al-Khatib et al., 2018; Hsia and Xiong, 2019). We first pick out three farthest modeling sites to predict the coordinates of point 0. Assuming points 1, 2, and 3 are selected, then we will remove them from the list of potential modeling sites that have never been used, so as not to select the duplicate modeling sites next time. Hence, the list will be updated to Adams et al. (2012), Sapp et al. (2016), Hsia and Xiong (2019).

Step 2: Train the models mentioned above to predict the target site.

Step 3: Termination judgment. If there are no unused potential modeling sites left, terminate. If the predicted site hits the target, terminate.

Step 4: Evaluate predicted site. If the predicted site is outside 35 mm of the target site, it is not credible, skip to step 6.

Step 5: Pick out a nearest site from the unused potential modeling sites if it is within 15 mm of the predicted site, then remove it from the list of unused potential modeling sites and skip to step 2. Otherwise, there is no unused potential modeling site that can replace the predicted site, turn to the next step.

Step 6: Pick out a site that is farthest from the geometric center of current modeling sites to obtain as much spatial information as possible, and remove it from the list of unused potential modeling sites and skip to step 2.

## Results

### Hits and Misses

**FIGURE 7:**
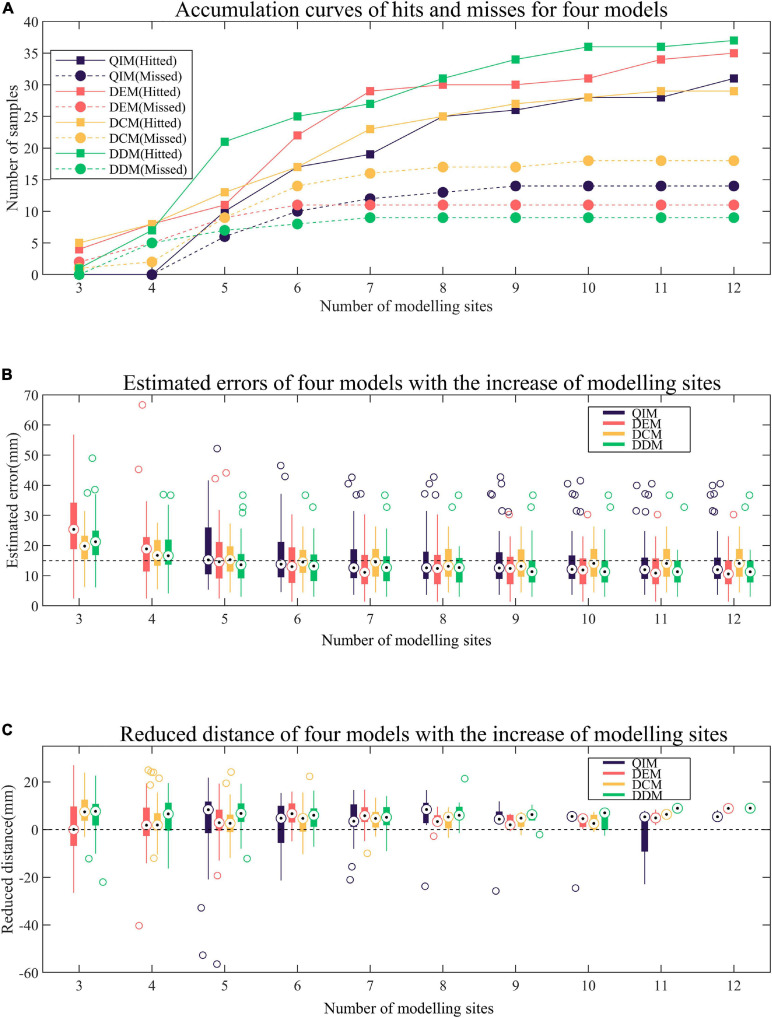
Comparison of four models. **(A)** Accumulation curves of hits and misses for four models. A hit occurs when the predicted site is within 15 mm of the target. **(B)** Estimated errors of four models with the increase of modeling sites. The estimated error is the distance between the predicted site and the target. **(C)** Reduced distance of four models with the increase of modeling sites. The reduced distance is equal to the minimum distance between the modeling sites and the target minus the estimated error.

A total of 47 target sites are used to evaluate the hit rate of four models. As Figure 7A shows, the proposed DDM presents with the most hits (37/47), the fewest misses (9/47), and one early termination. Here an early termination means that the reduction of estimated error is interrupted by lack of potential modeling sites. Then, slightly inferior to DDM, DEM performs with 35 hits, 11 misses, and one early termination. Finally, inferior to DDM and DEM, QIM and DCM achieve with 31 hits, 14 misses, one early termination and 29 hits, 18 misses, and no early termination, respectively. In addition, when the number of modeling sites is 5, DDM has much more hits than other models.

### Estimated Error

Figure 7B presents the trend of estimated errors of four models with the increase of modeling sites. It must be noted that for each target site, the estimated error remains unchanged after minimization. As can be observed from the figure, with the increase of modeling sites, the estimated errors of four models tend to decrease, especially when the number of modeling sites is less than 8 when most of the samples remained non-minimization (see Figure 7A). In terms of decline velocity of estimated error, DCM and DDM perform better than QIM and DEM when number of modeling sites is less than 5. Also, in terms of final estimated error, DEM and DDM perform better than QIM and DCM.

### Reduced Distance

The reduced distance is equal to the minimum distance between the modeling sites and the target site minus the estimated error, and a positive reduced distance indicates a reduction in the unknown range of the target by modeling and prediction. Figure 7C shows the reduced distances of four models with the increase of modeling sites. For each number of modeling sites, samples that have reached the minimum estimated error are not counted. As the figure shows, for different numbers of modeling sites, the mid-values of reduced distances of four models are almost positive, indicating that four models tend to reduce the unknown range of the target. When the number of modeling sites is 5, QIM has the largest mid-value of reduced distances and the corresponding hit rate also rises rapidly (see Figure 7A). However, due to the cumulative reduction of distances in the previous two rounds, DDM still has the highest hit rate.

### Target Range

**FIGURE 8:**
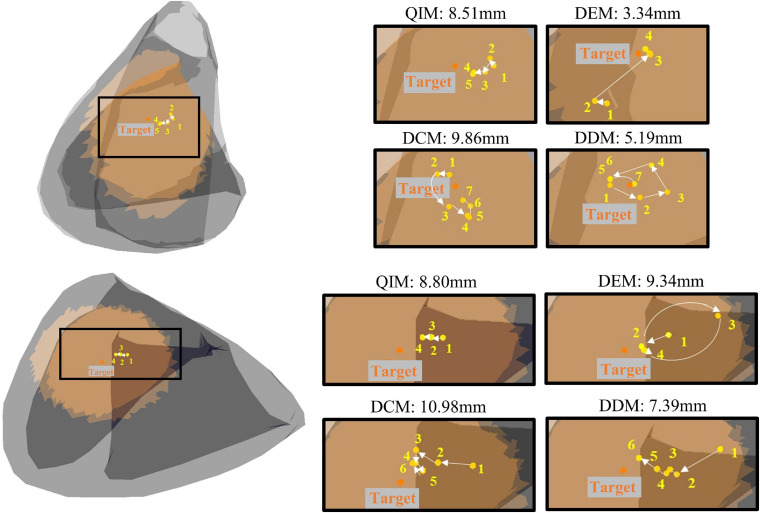
Two examples of predicted sites of four models with the increase of modeling sites. Orange dots mark the target, and yellow dots mark the predictions in the annotated order.

A total of 54 target sites are used to evaluate the efficiency of different models in narrowing down the target range. Figure 8 shows two examples of the predicted sites of four models with the increase of modeling sites. The initial target range (marked in orange in Figure 8) is defined by the maximum radius of adjacent sites, and the final target range is defined by the minimum estimated error. As the figure shows, the first target site has an initial radius of 32.92 mm; after modeling and prediction, the radius is reduced to 8.51, 3.34, 9.86, and 5.19 mm with 4 models, respectively. Similarly, the radius of the second target is reduced from 33.13 to 8.80, 9.34, 10.98, and 7.39 mm with 4 models, respectively.

Table 1 lists the statistical results of 54 target sites. Among the four models, DDM narrows down the target range most, from 27.62 ± 3.47 mm to 10.72 ± 6.22 mm, and DEM uses the fewest modeling sites (5.98 ± 2.49) to minimize the target range. In addition, t tests show that the estimation errors of DCM and DDM have a significant difference (*P* = 0.046), and the numbers of modeling sites of QIM and DEM, QIM and DDM have a significant difference (*P* = 0.007, *P* = 0.045), indicating that DCM has the worst estimated error and QIM used the most modeling sites.

**TABLE 1:** Comparison of four models in narrowing down the target range.

**Models**	**Radius of neighboring sites (mm)**	**Estimated error (mm)**	**Number of modeling sites used**
QIM	Mean: 27.62	Mean: 12.41	Mean: 7.28*^2,^ *^3^
	Std: 3.47	Std: 8.05	Std: 2.43
	Mid: 27.53	Mid: 10.05	Mid: 7
DEM	Mean: 27.62	Mean: 11.08	Mean: 5.98*^2^
	Std: 3.47	Std: 6.03	Std:2.49
	Mid: 27.53	Mid: 9.71	Mid: **5.5**
DCM	Mean: 27.62	Mean:13.03*^1^	Mean: 6.80
	Std: 3.47	Std: 5.67	Std: 2.47
	Mid: 27.53	Mid: 12.21	Mid: 6
DDM	Mean: 27.62	Mean: 10.72*^1^	Mean: 6.37*^3^
	Std: 3.47	Std: 6.22	Std: 2.22
	Mid: 27.53	Mid: **9.58**	Mid: 6

*^1^, *^2^, and *^3^ represent statistically significant differences between groups.

## Discussion

This work proposed a novel model for the localization of PVC target sites based on the mapping between the spatial domain and the morphological domain. In our study, the pacing sites are not as adjacent as those generated by clinical pace mapping, so we selected modeling sites from a larger range to predict the target site step by step. For inexperienced doctors, the results obtained by our method may provide a reference location, so that they can simply determine the most likely ablation site as soon as possible and shorten the mapping procedure. We compared our model with three existing models and found that the proposed model was slightly superior to other models by achieving the most hits, the smallest estimated errors, and the biggest reduced distances. Especially when the number of modeling sites is small, the advantages of our model are more obvious. By observation of Figure 7, it can be found that the proposed DDM tends to have more hits, smaller estimated errors, and bigger reduced distances than the other methods when the number of modeling sites is less than 6.

Then, as can be observed from Table 1, considering the minimum estimated error, DDM, and DEM perform better than QIM and DCM. Compared with DDM and DEM, QIM only uses the information of three-lead ECG, which may account for its less satisfying result. Though DCM also uses full information of 12-lead ECG, according to reference (Li et al., 2017), in contrast to Corr, E12 theoretically has no upper limit and, therefore, can provide better quantification of the morphology difference than Corr.

In addition, there is a certain relationship between DDM and DEM. In essence, DEM is to establish a scalar model through the relationship of the module length between dp and dw, while DDM directly uses the relationship between dp and dw to build a vector model. When the number of modeling sites is less than 5, the prediction effect of DEM is worse than that of DDM, which is likely due to the lack of direction information. However, when the number of modeling sites increases, the lack of direction information is gradually compensated by more and more complete distance network between points, and DEM achieves a similar result to DDM.

Finally, in terms of computational complexity, QIM, and DDM are simpler since the fitted models can be used for prediction directly, while DEM and DCM are more complex due to the additional search for optimal solution that can minimize the cost function *J*. Table 2 lists a summary comparison of four models.

**TABLE 2:** Summary comparison of four models.

**Model**	**Principles**	**Properties**	**Performance**
QIM	Using QRS-Ints as predictors	Less computation, using information of 3-lead ECG and containing direction information	Second least hits and second least reduced target range
DEM	Based on the relationship between distance and morphology difference	More computation, using information of 12-lead ECG and lack of direction information	Second most hits and second most reduced target range
DCM			Least hits and least reduced target range
DDM	Based on the mapping between spatial domain and morphological domain	Moderate computation, using information of 12-lead ECG and containing direction information	Most hits and most reduced target range

However, there still exist some limitations in this study. First, the way of picking out modeling sites is relatively random. Theoretically, the next modeling site should be the predicted result if it is reliable; otherwise, the next modeling site is selected by the doctor. Limited by the actual distribution of pacing sites, we take the second place and replace the predicted site with the nearest one among the unused potential modeling sites, which may cause the randomness in modeling site selection due to the different prediction results of four models, for example, when we design a fixed modeling site selection scheme, in which we choose a furthest site from the remaining potential sites in each round. Consequently, QIM, DEM, DCM, and DDM achieve 32, 35, 28, and 33 hits, respectively, indicating that the way of modeling site selection has a certain impact on the research results and the proposed DDM is more suitable for selecting modeling sites by referring to the predicted positions. Because collecting multiple-pace data in clinical practice will increase the risk of patients during operation, this kind of data is difficult to obtain. Therefore, we mainly used the data in the open database collected from three volunteers provided by Charles University in accordance with the strict experimental process. From the perspective of the number of individual patients, our sample is still relatively small, but the total number of test sites used in this paper is relatively large. In the future research, we can also consider the application of four methods to animal experimental data or retrospective clinical data analysis.

## Conclusion

To conclude, it is a desirable goal to develop an automated algorithm for the localization of PVC origins. This work provided a novel solution based on the mapping between spatial domain and morphological domain. It performs better with fewer modeling points and is expected to be used to predict the origin of ventricular activation in real-time and guide clinicians to focus on ablation targets.

## Data Availability Statement

The raw data supporting the conclusions of this article will be made available by the authors, without undue reservation.

## Ethics Statement

The studies involving human participants were reviewed and approved by the Ethical Committee of Charles University Hospital, Prague, Czechia. The patients/participants provided their written informed consent to participate in this study.

## Author Contributions

KH and JS: conceptualization, formal analysis, and methodology. CY: resources, supervision, and project administration. KH, YW, and GZ: software and visualization. KH: writing—original draft preparation. CY and GZ: writing—review and editing. KH and YW: revising and correcting. JS, KH, and CY: clinical interpretation and discussion of findings and their relevance. All authors contributed to the article and approved the submitted version.

## Conflict of Interest

The authors declare that the research was conducted in the absence of any commercial or financial relationships that could be construed as a potential conflict of interest.
